# Inflammation and LPS-Binding Protein Enable the Stimulatory Effect of Endotoxin on Prolactin Secretion in the Ovine Anterior Pituitary: Ex Vivo Study

**DOI:** 10.1155/2018/5427089

**Published:** 2018-08-14

**Authors:** Dorota Tomaszewska-Zaremba, Karolina Haziak, Monika Tomczyk, Andrzej P. Herman

**Affiliations:** Department of Animal Physiology, The Kielanowski Institute of Animal Physiology and Nutrition, Polish Academy of Sciences, 05-110 Jabłonna, Poland

## Abstract

Prolactin is a hormone that plays an important role in the regulation of many physiological processes including lactation, reproduction, fat metabolism, and immune response. The secretion of prolactin could be disturbed by an immune stress commonly accompanying infection. This study was designed to determine the influence of bacterial endotoxin—lipopolysaccharide (LPS)—on prolactin gene (PRL) expression and prolactin release from the ovine anterior pituitary (AP) explants collected from saline- and LPS-treated ewes in the follicular phase. The expressions of toll-like receptor 4 (TLR4) and proinflammatory cytokines interleukin- (IL-) 1*β*, IL-6, and tumor necrosis factor- (TNF-) *α* genes were also assayed. The results of the study showed that LPS stimulates prolactin secretion and IL-6 gene expression in the AP explants, but its action on lactotrophs depends on the immunological status of animal. It was demonstrated that an important role in enhancing the effect of LPS on the pituitary in the saline-treated ewes is played by LPS-binding protein (LBP)- “adapter molecule” for LPS binding to the cell surface receptor CD14 and then to TLR4. Also, it was found that bacterial endotoxin acting on the anterior pituitary cells may enhance prolactin secretion, and this effect of LPS could be mediated by IL-6 which is known as prolactin-releasing factor. Identification of the neuroendocrine and immune interactions in the regulation of prolactin secretion could be helpful in developing newer and more effective treatments for dysfunctions connected with disorders in this hormone secretion.

## 1. Introduction

Prolactin, a 200-amino acid peptide, is one of the most versatile hormones in the organism. It is secreted by glandular cells (lactotroph cells) from the anterior pituitary gland (AP). This peptide acts not only in endocrine but also autocrine and paracrine ways (as a growth factor, neurotransmitter, or immunomodulator) [1]. The release of prolactin is regulated by many factors. Dopamine is considered as the main negative regulator of its secretion; however, it could be also inhibited by acetylcholine, atrial natriuretic peptide, bombesin-like peptides, leukemia inhibitory factor, some prostaglandins, and cytokines such as IL-1*β* [1–3]. Although dopamine is known as the main prolactin inhibitory factor, the main prolactin-releasing factor has not been defined yet. However, the stimulatory effect on the prolactin secretion is exhibited by estradiol, oxytocin, gamma-aminobutyric acid, norepinephrine, serotonin, salsolinol, and IL-6 [1, 3–7]. Moreover, it was found that gonadotropin-releasing hormone (GnRH) may significantly stimulate prolactin secretion in the pituitary lactotrophs [8, 9].

Usually, prolactin is known as a hormone crucial for mammary gland functioning and so the regulation of lactation, but it is also involved in the regulation of numerous processes including reproduction, growth, development, fat metabolism, hair shedding, cellular differentiation, osmoregulation and immune response [1, 10]. Distortion of prolactin release is commonly accompanied by many diseases, such as infertility, impotence, osteoporosis, cancer, and also some autoimmune disorders, for example, systemic lupus erythematosus, rheumatoid arthritis, adjuvant arthritis, and cystic fibrosis [11]. Moreover, enhanced release of prolactin occurs during an immune stress induced by bacterial endotoxin [12, 13]. In the studies on animal model, immune stress is induced by intravenous injection of the bacterial endotoxin—lipopolysaccharide (LPS) [14]. It is a component of the outer membrane of gram-negative bacteria, and it is released into the circulation from replicating and dying bacterial cell [15]. Endotoxin stimulation of animal cells occurs through a signaling cascades with several proteins including CD14 protein, MD-2 protein, and LPS-binding protein (LBP), a necessary component of corresponding LPS receptor—Toll-like receptor 4 (TLR4) [16]. LPS associated with LBP, which is one of the acute-phase proteins, enters the bloodstream. Then, LPS-LBP complex binds to the CD14 protein, which is necessary for the activation of TLR4. Together CD14, MD-2, and TLR4 make up the cellular LPS specific receptor [17, 18]. After activation by endotoxin, TLR4 transduces its inflammatory signal through complex intracellular pathways, leading to the activation of transcription factors such as nuclear factor kappa-light-chain-enhancer of activated B cells (NF-*κ*B), c-Jun N-terminal kinase (JNK) and protein kinases p38, or inducing cell apoptosis [16–18]. TLR4 is not only present on the immune cells but its expression was also found in the AP cells [16, 19, 20]. It suggests that the changes in the secretion of pituitary hormone occurring during bacterial infection may be caused, at least partially, by LPS reaching the pituitary gland and directly affecting its secretory functions.

Therefore, the hypothesis of the present study was that LPS modulates prolactin secretion from the anterior pituitary explants collected from saline- and endotoxin-treated ewes in the follicular phase of the estrous cycle, and this action can be dependent upon the presence of LBP.

## 2. Materials and Methods

### 2.1. Ex Vivo Experiment

All procedures on animals were performed with the consent of the Local Ethics Committee of Warsaw University of Life Sciences-SGGW (Warsaw, Poland; authorization number 50/2013).

The ex vivo experiments were carried out on the tissues collected from 12 adults, 3-year-old Blackhead ewes in the follicular phase of the estrous cycle. For standardization of the experimental conditions, estrous cycles of ewes were synchronized by the Chronogest® CR (Merck Animal Health, Boxmeer, The Netherlands) according to the method described elsewhere [21].

The animals were divided into two subgroups: control (saline treated, *n* = 6) and LPS treated (*n* = 6). Ewes were injected into the jugular vein with an appropriate volume of LPS from *Escherichia coli* 055:B5 (400 ng/kg) (Sigma-Aldrich, St Louis, MO, USA) dissolved in saline (0.9% *w*/*v* NaCl) (Baxter, Deerfield, IL, USA). The maximum volume of injected LPS solution (10 mg/L) never exceeded 2.5 mL. The control group received the same volume of NaCl (based on their body weight). Three hours after the LPS or saline injections, all animals had rectal body temperature measured; it was 38.0 ± 0.3 and 39.6 ± 0.4°C for the saline- and LPS-treated groups, respectively. Three hours after the LPS or saline injections, all animals were euthanized by decapitation and collected anterior pituitaries were divided into 6 explants and transferred immediately to 24-well plates (Becton Dickinson Labware, Franklin Lakes, NJ, USA). The ex vivo incubation of the explants was performed in Medium 199 HEPES Modification (Sigma-Aldrich, St. Louis, MO, USA) with penicillin-streptomycin solution at a dose of 10 mL/L (Sigma-Aldrich, St. Louis, MO, USA) and incubated at 37°C, 87% O_2_, and 5% CO_2_. All tissues were preincubated for 2 h in 800 *μ*L of “pure” medium 199. During preincubation, every 30 min, the medium was changed 4 times for the fresh one to wash out blood and hormones remaining from pituitary fragments. Then, the explants collected from each saline- and LPS-treated ewe were divided into 6 parts and randomly assigned to one of the experimental groups as follows: I—control (“native”): incubated in “pure” medium 199; II—GnRH control: treated with GnRH (100 pmol/mL); III—LPS: treated with LPS (10 ng/mL); IV—LPS + GnRH: incubated in medium with both LPS (10 ng/mL) and GnRH (100 pmol/mL); V—LPS + LBP: treated with LPS (10 ng/mL) and LBP (120 ng/mL); VI—LPS + LBP + GnRH: treated with LPS (10 ng/mL), LBP (120 ng/mL), and GnRH (100 pmol/mL). After 4 h of incubation, all explants and media were frozen at −80°C until further assays.

### 2.2. Radioimmunoassay for Prolactin

The concentration of prolactin in medium was assayed by the RIA double-antibody method using specific anti-ovine-PRL and anti-rabbit-*γ*-globulin antisera as previously described [22]. The prolactin standard for iodination was obtained according to the method described elsewhere [23]. The sensitivity was 2 ng/mL; intra-assay and interassay coefficients of variation were 9 and 12%, respectively.

### 2.3. Relative Gene Expression Assay

NucleoSpin® RNA kit (MACHEREY-NAGEL GmbH and Co., Düren, Germany) was used to isolate the total RNA from the anterior explants. The purity and concentration of the isolated RNA were quantified spectrophotometrically with the use of NanoDrop 1000 instrument (Thermo Fisher Scientific Inc., Waltham, MA, USA). The integrity of isolated RNA was confirmed by electrophoresis with the use of 1% agarose gel stained with ethidium bromide. The Maxima™ First Strand cDNA Synthesis Kit for RT-qPCR (Thermo Fisher Scientific, Waltham, MA, USA) was used to perform cDNA synthesis. As a starting material for cDNA reversed transcription reaction synthesis, 1 *μ*g of total RNA was used. Real-time PCR was carried out with the use of the HOT FIREPol EvaGreen® qPCR Mix Plus (Solis BioDyne, Tartu, Estonia) and HPLC grade previously designed oligonucleotide primers (Genomed, Warszawa, Poland) (Table 1). One tube contained 4 *μ*L PCR Master Mix (5x), 14 *μ*L RNase-free water, 1 *μ*L primers (0.5 *μ*L each, working concentration was 0.25 *μ*M), and 1 *μ*L cDNA template. The tubes were run on the Rotor-Gene Q (Qiagen, Duesseldorf, Germany). The following protocol was used: 15 min in 95°C for activating Hot Start DNA polymerase and finally the PCR including 30 cycles at 95°C for 10 sec for denaturation, 20 sec in 60°C for annealing, and 10 sec in 72°C for extension. After the cycles, a final melting curve analysis under continuous fluorescence measurements was performed to confirm the specificity of the amplification. The relative gene expression was calculated using the comparative quantification option [24]—Rotor-Gene Q software (Qiagen, Dusseldorf, Germany). Three housekeeping genes were examined: glyceraldehyde-3-phosphate dehydrogenase (GAPDH), *β*-actin (ACTB), and histone deacetylase 1 (HDAC1). The mean expression of these three housekeeping genes was used to normalize the expression of the analysed genes. The results are presented in arbitrary units, as the ratio of the target gene expression to the mean expression of the housekeeping genes. For prolactin and TLR4 gene expression analyses, average relative quantity of gene expression in the control group of the anterior pituitary explants collected from saline-treated ewes was set to 1.0. In the case of all examined proinflammatory cytokine gene expression, average relative quantity of TNF gene expression in the control group of the anterior pituitary explants collected from saline-treated ewes was set to 1.0.

### 2.4. Data Analysis

The raw data, after passing the normality test, were subjected to repeated-measures three-way analysis of variance (ANOVA, GraphPad Prism, San Diego, CA, USA) followed by a post hoc Sidak's multiple comparison test. Statistical significance was established at *p* < 0.05.

## 3. Results

### 3.1. The Ex Vivo Effect of LPS on Prolactin Release

**Figure 1:**
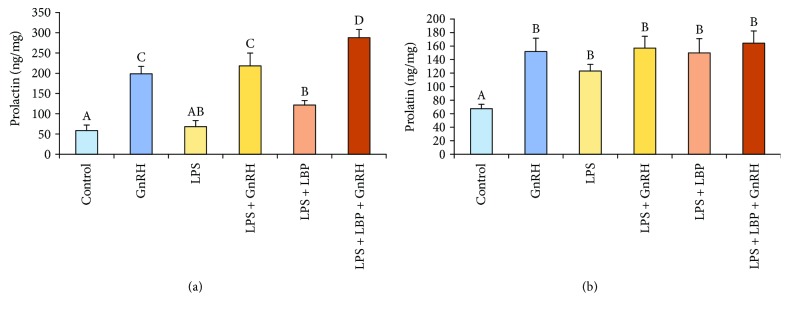
The effects of LPS, GnRH, and LBP on prolactin release from the AP explants collected from control (a) and LPS-treated (b) ewes. All data are presented as the mean (± S.E.M.). Different capital letters indicate significant (*p* < 0.05) differences according to a repeated-measures three-way analysis of variance (ANOVA, GraphPad Prism, San Diego, CA, USA) followed by a post hoc Sidak's multiple comparison test.

It was found that GnRH stimulated (*p* < 0.05) prolactin release in the explants from both saline- and LPS-treated ewes. In the explants collected from saline-treated animals, the addition of LPS stimulated (*p* < 0.05) prolactin release acting only together with LBP. On the other hand, the addition of LBP was not required for the LPS-dependent stimulation (*p* < 0.05) of prolactin release in the case of explants from endotoxin-treated animals. Moreover, it was found that explants from saline-treated ewes co-treated with GnRH, LPS, and LBP showed the highest release of prolactin among experimental groups. However, this additive effect of concomitant treatment with GnRH, LPS, and LBP did not occur in the case of explants from LPS-treated animals (Figure 1).

### 3.2. The Ex Vivo Effect of LPS on *PRL* Gene Expression in AP Explants

It was determined that GnRH enhanced (*p* < 0.05) *PRL* gene expression in all treated AP explants. In the explants collected form saline-treated ewes, LPS-dependent increase in *PRL* gene expression was found only in the group concomitant treated with LPB. The presence of LPB was not required for stimulatory (*p* < 0.05) effect of LPS on *PRL* gene expression in the explants from endotoxin-treated animals. It was found that the group of AP explants collected from LPS-treated ewes concomitant treated with GnRH and LPS as well as GnRH, LPS, and LBP were characterized by significantly (*p* < 0.05) higher expression of *PRL* gene compared with the groups treated only with GnRH or LPS or LPS and LBP (Table 2).

### 3.3. The Ex Vivo Effect of LPS on *TLR4, IL-1β, IL-6, TNFα* Gene Expression in AP Explants

No effect of the treatments on the gene expression of *TLR4, IL-1β*, and *TNFα* was found. On the other hand, in AP explants from saline-treated ewes ex vivo treated with both LPS and LBP, an increase (*p* < 0.05) of *IL-6* gene expression was found. Whereas in AP explants from endotoxin-treated animals, LPS-dependent increase (*p* < 0.05) in the level of *IL-6* mRNA level was noted regardless of addition of LPS alone or together with LBP (Table 3).

## 4. Discussion

Being an important indicator of stress is one of the many attributes of prolactin. Its increased secretion during inflammation is often considered to be the effect of stress caused by the activation of immune system [25]. In previous *in vivo* studies, it was shown that immune stress caused by peripheral administration of endotoxin stimulated prolactin release in sheep [12, 13]. In anestrous ewes, prolactin secretion was increased after intravenous administration of lipopolysaccharide and was associated with increased transcription of mRNA encoding prolactin in the AP [13]. It is considered that inflammation induced by bacterial endotoxin stimulates the prolactin secretion through activation of different processes at the hypothalamic level. As suggested in the study on rats, stress-induced prolactin release may be caused by corticotropin-releasing factor (CRF) because the blockade of its type I receptor results in a significant decrease in the circulating concentration of prolactin [26]. Moreover, the elevated level of prolactin in the blood during the immune stress could be the result of the central action of proinflammatory cytokines—central treatment with IL-1*β* significantly increased the prolactin secretion in rat [27]. Also, the changes in the prolactin secretion can be caused by the central action of LPS. In the *in vivo* study carried out on male rat, it was shown that intracerebroventricular (icv) administration of LPS into the third ventricle of the brain caused an increase in the serum prolactin levels at 12 and 24 h after injection, although this increase was significant only at 24 h [28]. It is worth mentioning that more and more researchers suggest that during the infection, LPS, particularly its component, lipid A, crosses the blood-brain barrier and penetrates brain parenchyma [29]; therefore, its direct action on the centrally regulated processes could not be marginalized.

However, our study suggests that inflammation may influence the secretion of prolactin through processes occurring at the pituitary level. In our ex vivo experiment, it was demonstrated that LPS affects the PRL gene expression and prolactin release from AP explants collected from both saline- and LPS-treated ewes. However, it is suggested that this stimulatory action of LPS on the prolactin secretion, at least partially, results from the action of locally synthesized IL-6. A significantly increased IL-6 gene expression was found in LPS-treated explants. This finding is in agreement with the previous study in which lipopolysaccharide acting via TLR4 stimulated the folliculostellate cells to release such proinflammatory cytokines as IL-6 [20]. It was suggested that the increase in the prolactin secretion during inflammation may be induced by IL-6 affecting the secretory activity of the pituitary by a direct stimulation of pituitary lactotrophes to release prolactin [6, 7]. Moreover, receptors of IL-6 have been detected in the brain and in the pituitary gland and it was confirmed that this interleukin stimulates prolactin secretion by the anterior pituitary gland [30]. The basal release of prolactin was inhibited by a polyclonal antiserum of rat IL-6, showing the involvement of IL-6 in the prolactin secretion [31]. However, in our study, it was demonstrated that increased prolactin secretion occurring in line with immune challenge, at least partly, may result from the direct action of circulating bacterial endotoxin on the pituitary lactotrophs. Although, basing on our data, it is incredibly difficult to judge which type of anterior pituitary cells is a main source of this cytokine. It could be supposed that a profound amount of pituitary IL-6 is released from folliculostellate cells. It was previously described that folliculostellate cells in the pituitary gland are the source as well as the target of proinflammatory cytokines, such as IL-1, IL-6, and TNF*α* [32, 33], and they may act in paracrine or autocrine pathways on the other pituitary cells. The lack of effect of the LPS treatment on the gene expression of IL-1*β* and TNF*α* in the AP explants from control (saline treated animals) and LPS (LPS treated animals) groups is surprising: however this may result from autocrine and paracrine action of IL-6 on the pituitary cells and suppression of these cytokines transcription. It is known that IL-6 is a unique pleiotropic cytokine exhibiting both pro- and anti-inflammatory properties and, among other actions, inhibiting both IL-1*β* and TNF*α* production [34].

The ability of LPS to interfere with the pituitary cells is possible due to the expression of its corresponding receptor TLR4 in this gland. The presence of TLR4 receptors in AP was also confirmed in our previous study on anestrous ewes [16] as well as in ex vivo experiment on AP explants from ewes in follicular phase [19]. The current ex vivo study confirms the presence of TLR4 in AP explants both from saline- and LPS-treated animals, but any of experimental treatments influenced the gene expression of this receptor. It was shown that LPS-dependent stimulation of prolactin secretion from AP explants collected from intact, saline-treated animals required the presence of LBP, whereas in tissues collected from the animals subjected to the immune stress, the addition of this protein was not needed for LPS action. This fact once again supports the result of our previous study indicating that the activity of LPS on the ovine pituitary cells from intact animals requires LBP presence [19]. The ability of LBP to enhance the response of LPS was also demonstrated in both *in vivo* and *in vitro* studies [35, 36]. However, under some circumstances, LPS could stimulate TLR4 alone but the spontaneous diffusion of LPS monomers to the cellular-binding site is very slow, and transfer by LBP enhances the immune response to LPS up to 1000-fold *in vitro* [36]. It is worth mentioning that the biological role of LBP is concentration dependent. High LBP concentration can inhibit LPS bioactivity *in vitro* and *in vivo* [37]. The fact that in the explants from LPS-treated animals the presence of LBP was not necessary suggests that during endotoxin-induced immune stress the LBP level in the pituitary gland was enough to enable action of LPS in AP explants.

Our results also confirm a stimulatory effect of GnRH on prolactin secretion both in saline- and LPS-treated animals. In the experiments on human pituitary monolayer cell cultures, it was also found that GnRH stimulated prolactin secretion by direct action on pituitary cells *in vitro* [38]. Our ex vivo study was performed on the explants collected from animals during reproductive season which could be of profound importance for stimulatory effect of GnRH. It was previously described that in photoperiodic species, GnRH stimulates prolactin release from lactotrophs only during the breeding season [9]. It is worth noticing that although both LPS and GnRH generally stimulate prolactin secretion, PRL gene expression in our study was significantly higher in the groups of explants concomitant treated with LPS and GnRH in comparison with the groups in which LPS or GnRH were added alone. This suggests that the same biological stimulatory effect of these treatments may be achieved by the different cellular pathways.

## 5. Conclusions

Summarizing, it was shown that LPS stimulates prolactin secretion in AP explants and this effect, at least partially, could be mediated by IL-6 which gene expression in the explants was elevated after endotoxin treatment. However, this action of LPS on the pituitary seems to be dependent on the immunological status of the animal. Also, it was demonstrated that an important role in enhancing the effect of LPS on the pituitary in the saline-treated ewes plays LPS-binding protein—an “adapter molecule” for LPS-binding to the cell surface receptor CD14 and then to TLR4.

## Figures and Tables

**Table 1:** Specific primers used in real-time PCR analysis to determine the expression of housekeeping genes and genes of interest.

Gene	GeneBank acc. number	Amplicon size (bp)	Sequence 5′→ 3′	
			Forward	Reverse
*GAPDH^a^*	NM_001034034	134	AGAAGGCTGGGGCTCACT	GGCATTGCTGACAATCTTGA
*ACTB^a^*	U39357	168	CTTCCTTCCTGGGCATGG	GGGCAGTGATCTCTTTCTGC
*HDAC1^a^*	BC108088.1	115	CTGGGGACCTACGGGATATT	GACATGACCGGCTTGAAAAT
*PRb^b^*	NM_001009306	131	CCTCTCCTCGGAAATGTTCA	AGGACTTCATGGTGGGTCTG
*TLR4^c^*	AY957615	117	GGTTCCCAGAACTGCAAGTG	GGATAGGGTTTCCCGTCAGT
*IL1B^a^*	X54796.1	137	CAGCCGTGCAGTCAGTAAAA	GAAGCTCATGCAGAACACCA
*IL6^a^*	NM_001009392.1	165	GTTCAATCAGGCGATTTGCT	CCTGCGATCTTTTCCTTCAG
*TNF^a^*	NM_001024860	153	CAAATAACAAGCCGGTAGCC	AGATGAGGTAAAGCCCGTCA

References: ^a^[39]; ^b^[21]; ^c^[40].

**Table 2:** The effects LPS, GnRH, and LBP on the relative gene expression of PRL in the AP explants.

Gene	Animals	Anterior pituitary explants					
		Control	GnRH	LPS	LPS + GnRH	LPS + LBP	LPS + LBP + GnRH
*PRL*	Saline-treated	1 ± 0.1^A^	2.5 ± 0.2^BC^	1.3 ± 0.3 ^AB^	2.9 ± 0.3^C^	2.8 ± 0.1^C^	5.6 ± 0.3^D^
	LPS-treated	4.1 ± 0.4^A^	5.7 ± 0.2^B^	8.2 ± 0.3^C^	10.1 ± 0.2^D^	7.4 ± 0.4 ^C^	9.9 ± 0.8 ^D^

All data are presented as the mean (± S.E.M.). Different capital letters indicate significant (*p* < 0.05) differences according to repeated-measures three-way analysis of variance (ANOVA, GraphPad Prism, San Diego, CA, USA) followed by a post hoc Sidak's multiple comparison test. Gene expression data were presented as normalized to the control of saline-treated group.

**Table 3:** The effects LPS, GnRH, and LBP on the relative gene expression of TLR4 and proinflammatory cytokines in the AP explants.

Gene	Animals	Anterior pituitary explants					
		Control	GnRH	LPS	LPS + GnRH	LPS + LBP	LPS + LBP + GnRH
*TLR4*	Saline treated	1 ± 0.1^A^	0.9 ± 0.2^A^	1 ± 0.1^A^	0.9 ± 0.1^A^	1 ± 0.1^A^	0.8 ± 0.1^A^
	LPS treated	1.1 ± 1.5^A^	1.4 ± 2.2^A^	1.2 ± 2.8^A^	1.3 ± 1.9^A^	1.1 ± 1.2^A^	1.3 ± 5^A^

*IL1B*	Saline treated	2.1 ± 0.3^A^	2.5 ± 0.3^A^	2.3 ± 0.2^A^	2.6 ± 0.5^A^	1.7 ± 0.3^A^	2.6 ± 0.4^A^
	LPS treated	2.3 ± 0.4^A^	2.5 ± 0.2^A^	2.9 ± 0.2^A^	2.7 ± 0.2^A^	2.9 ± 0.2^A^	2.9 ± 0.2^A^

*IL-6*	Saline treated	7.4 ± 0.9^A^	7.1 ± 1.2^A^	9.3 ± 0.5^A^	8.1 ± 0.7^A^	13.9 ± 1.6^B^	13.8 ± 1.7^B^
	LPS treated	18.4 ± 1.5^A^	17.3 ± 2.2^A^	33.5 ± 2.8^CD^	24.9 ± 1.9^B^	27.1 ± 1.2^BC^	36.1 ± 5^D^

*TNF*	Saline treated	1 ± 0.2^A^	0.9 ± 0.3^A^	1.4 ± 0.2^A^	1.5 ± 0.3^A^	1.1 ± 0.1^A^	1.1 ± 0.2^A^
	LPS treated	1.1 ± 0.2^A^	1.2 ± 0.1^A^	1.4 ± 0.1^A^	1.3 ± 0.1^A^	1.2 ± 0.1^A^	1.2 ± 0.1^A^

All data are presented as the mean (± S.E.M.). Different capital letters indicate significant (*p* < 0.05) differences according to repeated-measures three-way analysis of variance (ANOVA, GraphPad Prism, San Diego, CA, USA) followed by a post hoc Sidak's multiple comparison test. Gene expression data were presented as normalized to the control of saline-treated group.

## Data Availability

The data used to support the findings of this study are available from the corresponding author upon request.
